# Medical-Grade ECG Sensor for Long-Term Monitoring

**DOI:** 10.3390/s20061695

**Published:** 2020-03-18

**Authors:** Aleksandra Rashkovska, Matjaž Depolli, Ivan Tomašić, Viktor Avbelj, Roman Trobec

**Affiliations:** 1 Department of Communication Systems, Jožef Stefan Institute, Jamova cesta 39, 1000 Ljubljana, Slovenia; matjaz.depolli@ijs.si (M.D.); viktor.avbelj@ijs.si (V.A.); roman.trobec@ijs.si (R.T.); 2 Division of Intelligent Future Technologies, Mälardalen University, Högskoleplan 1, 721 23 Västerås, Sweden; ivan.tomasic@mdh.se

**Keywords:** electrocardiography, body ECG sensor, differential lead, mobile health, medicine, sport, veterinary

## Abstract

The recent trend in electrocardiogram (ECG) device development is towards wireless body sensors applied for patient monitoring. The ultimate goal is to develop a multi-functional body sensor that will provide synchronized vital bio-signs of the monitored user. In this paper, we present an ECG sensor for long-term monitoring, which measures the surface potential difference between proximal electrodes near the heart, called differential ECG lead or differential lead, in short. The sensor has been certified as a class IIa medical device and is available on the market under the trademark Savvy ECG. An improvement from the user’s perspective—immediate access to the measured data—is also implemented into the design. With appropriate placement of the device on the chest, a very clear distinction of all electrocardiographic waves can be achieved, allowing for ECG recording of high quality, sufficient for medical analysis. Experimental results that elucidate the measurements from a differential lead regarding sensors’ position, the impact of artifacts, and potential diagnostic value, are shown. We demonstrate the sensors’ potential by presenting results from its various areas of application: medicine, sports, veterinary, and some new fields of investigation, like hearth rate variability biofeedback assessment and biometric authentication.

## 1. Introduction

Surface electrocardiogram (ECG) is cardiac electrical activity recording obtained with electrodes placed on the skin. An ECG showing both atrial and ventricular activity was first measured by Willem Einthoven at the beginning of the 20th century with his invention—the string galvanometer [1]. The whole ECG machine weighed about 300 kg. Today, a wide range of ECG devices are used in medicine: 12-lead ECG, multichannel ECG (MECG), Holter monitor, implantable loop recorder (ILR), and some others. The golden standard is the well-known 12-lead ECG, where wires are connected to electrodes placed on 10 locations on the body [2]. The MECG body surface mapping systems measure potentials simultaneously on more than 10 positions on the body [3]. The number of leads in MECG ranges usually from 20 to hundreds. The standard 12-lead ECG and the MECG systems are mostly intended for short-term monitoring in a resting position in a clinical environment. For ambulatory and long-term ECG monitoring, the most frequently utilized option is still the Holter monitor introduced by Norman J. Holter in 1961 [4].

The Holter monitors are smaller devices compared to standard 12-lead ECG device, but are still using wires to connect the electrodes with the recording machine. They have been the sole standard for monitoring ECG outside of hospitals since their invention in the sixties. Although these monitors have evolved into smaller and more powerful devices for recording high-quality single or multi-lead ECG, they cause discomfort for the patients because the device needs to be carried on the body with all the cabling. Furthermore, they still have limited duration of the recordings up to a maximum of 14 days. On the other hand, the (wireless) ILRs are lightweight devices (usually only around 17 g weight) implanted under the skin [5] and provide comfortable long-term ECG monitoring for up to several years. However, the ILRs are invasive devices and their capacity for ECG recording is limited to several recordings, each with a duration of a couple of minutes. Consequently, if the patient equipped with the device does not visit a medical office to retrieve the measurements in a timely manner, older recordings are overwritten.

The provision of mobile health (mHealth) services, like patient monitoring in hospitals [6], remote medical support, or monitoring during sport activities, requires for these ECG devices to allow greater patient mobility than the Holter monitor [7,8,9] and additionally provide wireless transmission of the data from the device to a nearby personal terminal (smartphone, tablet) connected to the Internet [10]. Motivated by these challenges, we have envisioned to design a monitoring system for synchronous measurement of vital bio-signs [11,12]. Our ultimate goal was to combine minimal number of body sensors with different functions into in a single one, i.e., to develop a multi-functional body sensor. Namely, our long-term experience with MECG devices has shown us that a significant amount of information about vital functions, including ECG, can be obtained from the electric potential between two neighboring MECG electrodes. However, a conductive path (a wire and some electronics) is still needed between the electrodes in order to measure the voltage, i.e., the electric potential difference. Using just a single conductive path to connect proximal electrodes near the heart, we can non-invasively obtain single-channel bipolar ECG, called differential ECG lead or differential lead (DL). An improvement from the user’s perspective—immediate access to the measured data—is also envisioned into the design. Appropriate placement of such device on the body near the heart could provide high-quality ECG recordings appropriate for medical use. Compared to the Holter monitor and ILR, our solution is situated somewhere in between. It is wireless, as the ILR, but costs less; as the Holter monitor, it is non-invasive and provides good visibility of all electrocardiographic waves. The latter is important, since ILRs often record ECGs where the P waves are not sufficiently visible.

In this paper, we present a wireless ECG sensor for long-term monitoring—its design, intended use, and various areas of its application. We refer to the sensor as sensor for personal cardiac monitoring (PCARD) or PCARD sensor. The sensor has been certified according to the Medical Devices Directive MDD 93/42/EEC [13] as a class IIa medical device and is available on the market under the trademark Savvy ECG (http://savvy.si/).

More details about body sensors and electrocardiography from the aspect of wireless and mobile ECG monitoring can be found in our recent monograph [9]. Additionally to the monograph, in this paper, we provide several novelties. First, we present detailed hardware and firmware design of the particular ECG sensor. Next, we make an extended comparison with related devices regarding the design concept and intended use. Finally, we demonstrate the sensors’ potential by presenting results from its various areas of application: medicine, fitness, veterinary, and some new fields of investigation, like hearth rate variability biofeedback assessment and biometric authentication.

## 2. Sensor Design

**Figure 1:**
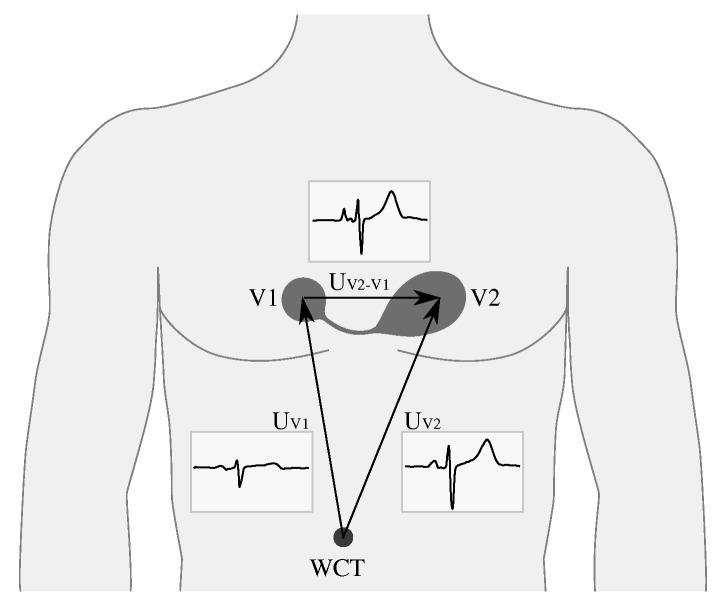
A differential lead from the positions of the V1 and V2 electrodes of a multi-channel ECG, with the corresponding voltages $U_{V1}$ and $U_{V2}$ and the resulting differential ECG denoted by $U_{V2-V1}$.

The idea for a wireless ECG sensor is based on the notion of differential lead or differential ECG, which can be measured as a potential difference between two channels in an MECG or a standard 12-lead ECG. In MECG, as well as in the standard 12-lead ECG, the leads are referenced to the Wilson central terminal (WCT). In this case, if the electrodes of the body sensor are positioned as two multi-channel electrodes, then the ECG signal is equal to the algebraic voltage difference of the two multi-channel leads. Similarly, if we position the electrodes on the sensor as the electrodes V1 and V2 in a 12-lead ECG, the differential lead is equivalent to the algebraic difference of the voltages in V1 and V2, as presented in Figure 1. The concept of a single unit is viable because the electrodes of a differential lead can be in close proximity. However, by increasing the proximity, the ECG signal becomes smaller while the noise level remains at the same level. Therefore, the electrodes should be kept at a distance that can provide reliable signal with satisfactory signal-to-noise ratio. The sensor, complemented with additional electronics, like low-power radio, processing unit, and battery, can be made lightweight in design, which allows for unobstructed every-day use.

The initial prototype of the wireless body sensor (WBS) was designed to measure ECG [14]. It was powered by a coin battery, and included a low power micro-controller and 2.4 GHz radio transceiver. The design was then improved to include a rechargeable battery and Bluetooth Low Power (BLE) radio transceiver for communication. For the measurement to start, the WBS is first attached to the skin by using self-adhesive electrodes. The raw signal is measured as a difference between the electrical potentials of the electrodes. An internal clock triggers the sampling and conversion of the analogue signal into a 10-bit digital sample. The signal is then streamed to a personal digital assistant (PDA), like a smartphone or a tablet, through a BLE connection. On the PDA, additional analysis can be performed and the signal can be visualized in real time [12]. For a satisfactory signal-to-noise ratio and minimal discomfort for the wearer, the optimal distance between the electrodes is experimentally found to be about 8 cm. As a compromise between sustainable power consumption and acceptable measurement quality, we selected 125 samples/s as the default signal sampling rate. If required, for example, for recording ECG of an infant, the sampling rate can be increased up to 1000 samples/s. Furthermore, the device could be also made multi-functional by incorporating additional sensors, like temperature sensor and accelerometer. The ECG potential difference on the body surface could also indirectly provide information on other vital functions, like respiration, by a customized analysis of the ECG signal [15]. Moreover, the standard 12-lead ECG can be synthesized from the measurements of three WBSs [16,17].

**Figure 2:**
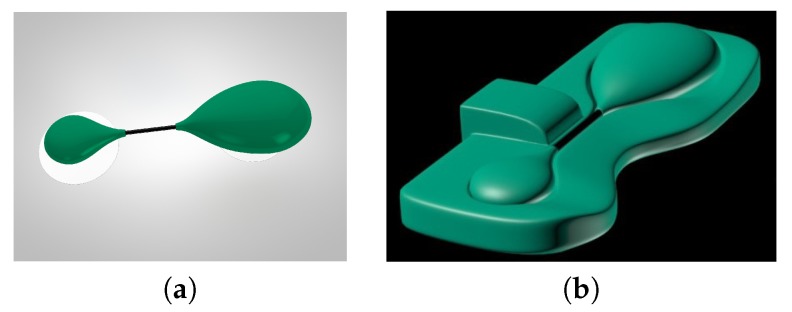
Savvy™ sensor (**a**) the wearable body sensor in bio-compatible housing with two self-adhesive electrodes attached; (**b**) the body sensor placed in the charging dock.

In time, the sensor has evolved into a more flexible and lightweight design that allows for unobtrusive long-term mobile health monitoring and low-cost implementation with an appropriate casing. The sensor as such is already produced as the commercial device Savvy™ shown in Figure 2. With a single charge of the built-in battery, the sensor can record ECG continuously up to 10 days. In the following, we present the sensor design, given by schematic representation of its hardware and firmware components.

### 2.1. Hardware

**Figure 3:**
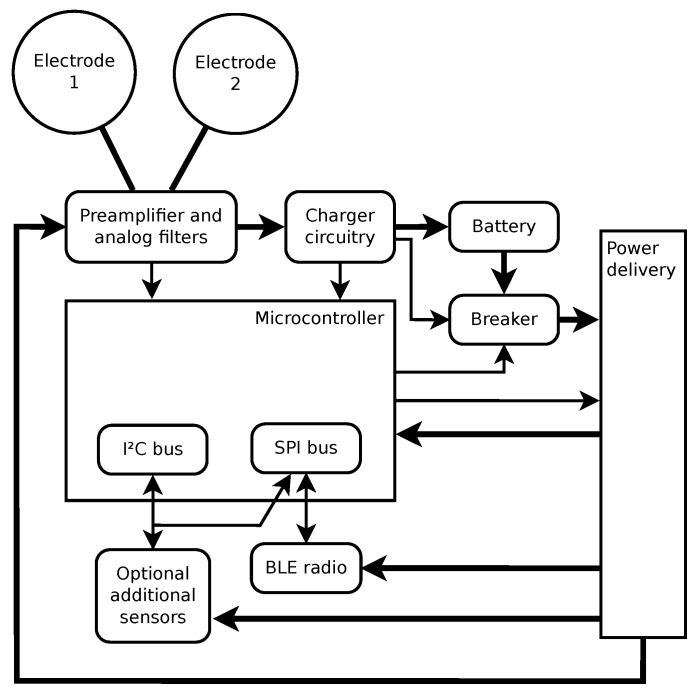
Hardware block diagram of the PCARD sensor. Thicker lines represent power delivery, while thinner lines represent signal transfer.

In Figure 3, the hardware block diagram of the ECG sensor is shown. Major hardware building blocks are identified:

- Electrodes 1 and 2—These two dual-purpose electrodes are the only physical interface with the outside world. They are used to either electrically connect the sensor to the body or to the external battery charger.
- Rechargeable battery.
- Preamplifier and analog filters—this circuitry takes the input signal and converts it to appropriate voltage levels to be detected by the micro-controller, applies the radio frequency low-pass filtering, ECG band-pass filtering, and provides the required input impedance for the electrodes to interface with the human body. Within this circuitry, the lines from the electrodes are also diverted to the charger circuitry.
- Charger circuitry—detects when the external battery charger is connected to the electrodes, and implements proper battery charging with hardware overcharge protection and circuit breaker resetting.
- Breaker—used to isolate all other circuitry from the battery to minimize power usage when the sensor is stored for a longer period of time.
- Micro-controller—the brains of the device, which provides ADC (analog to digital conversion) of input signals, communication with additional sensors (the sensors not embedded to the micro-controller), setting of the circuit breaker, and communication with the BLE radio transceiver.
- Optional supplementary sensors—Currently, the supplementary sensors include a thermometer and an accelerometer. They are optional in the sense that they only represent an additional functionality, which is not always required. For example, they are absent in the first production series of the sensor, which aims to provide a simple ECG measurement device.
- BLE radio—the final block of hardware used to connect to a device (like a smartphone) that will be used for data storage and control of the sensor.
- Power delivery—circuitry that enables the micro-controller to selectively deliver power to other building blocks. It is used to lower the power consumption while the sensor is not active.

The wearable sensor implements the following functionalities through its hardware and firmware:

- Data sampling—the process of converting the analog input signal into digital form by sampling it in regular intervals, for example, 125 times per second. It starts with the analog circuitry that converts the measured quantity into voltage on a predefined range for the ADC to measure. Then, it continues with the micro-controller that takes samples through its built-in ADC and stores them into a memory buffer, where they wait for further transfer.
- Data transfer—the process of transferring the collected data from the micro-controller to the smartphone. Samples are transmitted in bulk by the micro-controller over a standard SPI (Serial Peripheral Interface) to the chip that contains radio transceiver. The wireless transfer is then conducted by using the BLE protocol. More precisely, a custom wireless protocol built on top of BLE is used as the communication protocol between the sensor and the controlling device.
- Remote control and monitoring—provides the user interface towards the sensor through its BLE connectivity. There are several parameters and settings that may be defined by the user and also parameters that should be monitored to assure the required quality of service. Examples are sensor battery level and sampling rate selection.

### 2.2. Firmware

**Figure 4:**
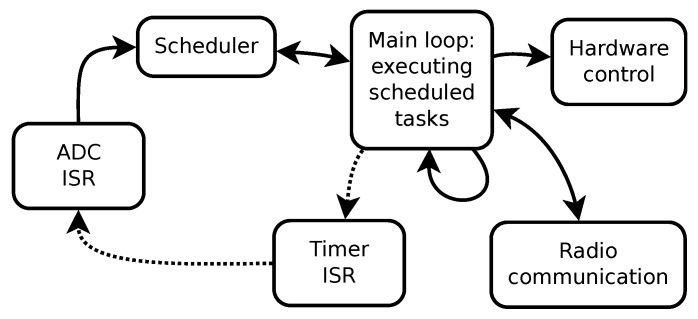
Firmware block diagram of the PCARD sensor. Full lines represent direct connections, while dotted lines represent indirect influence.

Figure 4 shows a block diagram of the firmware functionality. Each displayed block can be traced back to a function or a set of associated functions in the source code. The main loop runs from power on until power off and controls all three main elements: hardware, radio, and scheduler. First and foremost, the main loop implements the wireless communication protocol between the firmware and the remote device. The protocol comprises listening for commands from, and transmitting the data to, the remote device. The main loop executes the tasks scheduled by the scheduler. For data sampling, the main loop sets up a timer to periodically generate interrupts. With each timer interrupt, the timer interrupt service routine (ISR) is executed asynchronously. The ISR sets up the hardware for data sampling, i.e., the ADC of the micro-controller. After the asynchronously executed sampling, an ADC interrupt is generated by the ADC. This interrupt is handled by the ADC ISR, which caches the read sample. When enough samples have been collected, the ADC ISR bundles them into a single packet and attaches them to a task that is enqueued in the scheduler’s queue. The data are transferred to the radio when a new iteration of the main loop is started and the enqueued task is executed. A custom communication protocol defines the number of samples per packet, other contents of individual packets, and the rules for handling packets. The other job of the main loop is to control the rest of the hardware. This includes low level radio communication, setup of the radio and transfer of protocol parameters, continuous battery level control, and the control of the measurement and device power states.

### 2.3. Related Sensors

**Figure 5:**
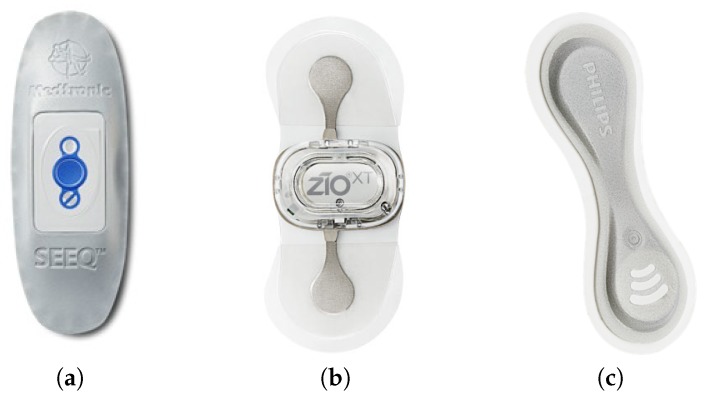
(**a**) SEEQ™ sensor by Medtronic, Inc.; (**b**) ZIO^®^ XT Patch by iRhythm Technologies, Inc.; (**c**) Philips biosensor.

Related sensors were identified among candidates that have similar characteristics as the PCARD sensor, especially regarding safety and performance, and additionally, are declared as CE-marked and/or FDA-approved body patch ECG devices. We compare the PCARD sensor with three competitive devices: the SEEQ™ sensor by Medtronic, Inc. (http://www.medtronicdiagnostics.com), the ZIO^®^ XT Patch by iRhythm Technologies, Inc. (http://www.irhythmtech.com) and the wearable biosensor by Philips (https://www.usa.philips.com/healthcare/clinical-solutions/early-warning-scoring/wireless-biosensor), shown in Figure 5.

Compared to the other devices, the PCARD sensor is reusable because it has a rechargeable battery, thus providing the opportunity for longer measurements. Skin sensitivity (skin irritation) to the compounds of the self-adhesive electrodes has been found to be one of the main problems with prolonged ECG measurements [18,19]. However, the PCARD sensor can partially alleviate this problems with the use of multiple pairs of disposable self-adhesive electrodes and re-positioning of the PCARD sensor away from the areas with irritated skin. Furthermore, the PCARD sensor coupled with a PDA, like a smartphone, constitutes an ECG monitoring system for long-term monitoring, alongside providing real-time visualisation of the ECG measurements. On the other hand, the SEEQ™ sensor is “single-use”, with the maximal recording period of up to 7.5 days per use. The recording period can be prolonged up to 30 days by deploying up to three additional sensor units. However, the data from the SEEQ™ sensor can be transmitted in real time to the company’s network via special transmitter device. In this case, the response time depends on the data processing time of their monitoring center. Similarly to the SEEQ™ sensor, the ZIO^®^ XT patch is used only once for up to 14 days. After that, it needs to be returned to the company by post, which means that all the processing is done offline, without any visualization of the ECG during measurement. In addition, the Philips biosensor is meant to be used for up to four days without repositioning and then recycled (the available information specifies it is a “single-use, single-location” device). The data are automatically and continuously gathered; however, the processing is done in the Cloud which Philips calls IntelliVue Guardian Software. The sensor uses a “relay” (a device that is similar to a smartphone with a case and a wall mount holder) to transfer the data to the Cloud. The patients need to be within 10 m of the relay or carry the relay device with them, when they would move beyond the stationary coverage area. Additionally to measuring ECG, the Philips biosensor is also equipped with an accelerometer and a thermistor for body temperature measurement.

## 3. Intended Use

**Figure 6:**
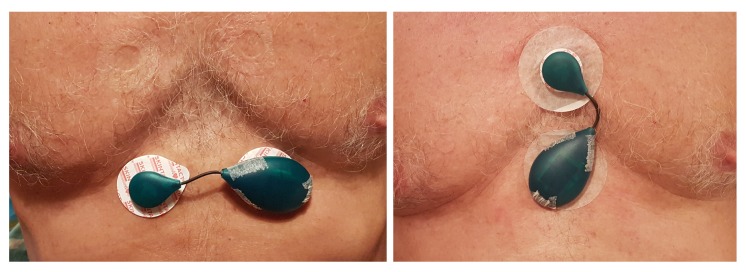
ECG sensors fixed with self-adhesive electrodes in two often used positions: horizontal (**left**) and vertical (**right**).

**Figure 7:**
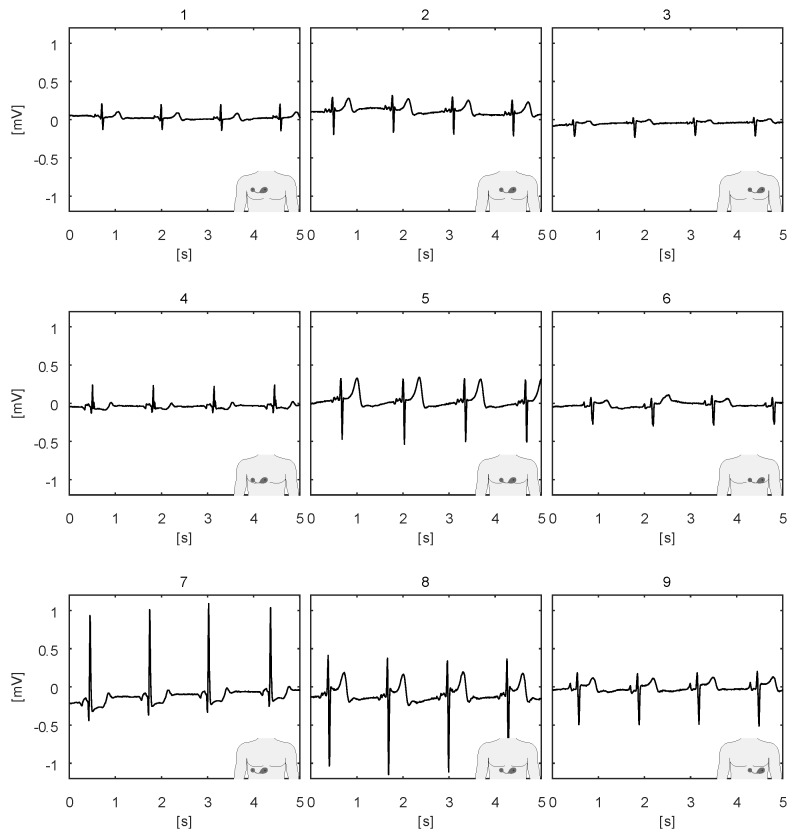
Raw ECG signals recorded from the ECG sensor in positions 1 to 9, obtained by translating the sensor from the horizontal position shown in Figure 6.

**Figure 8:**
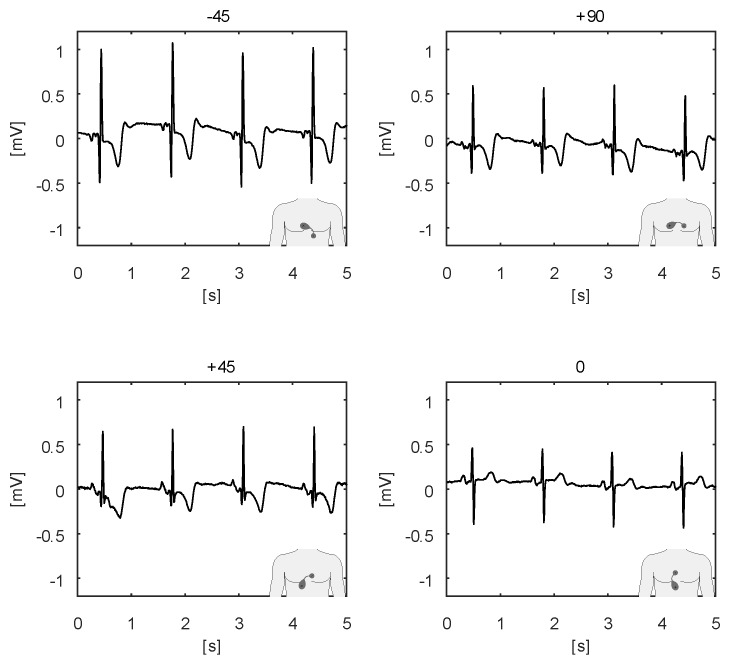
Raw ECG signals recorded in four positions, obtained by rotating the sensor from the vertical position shown in Figure 6, for $-45^{\circ }$, $45^{\circ }$, $90^{\circ }$, and $0^{\circ }$.

The ECG body sensor offers a novel type of electrocardiographic data compared to the standard 12-lead ECG [20,21]. The wireless ECG body sensor does not have a fixed position on the body as the 12-lead ECG apparatus. Therefore, its output is not comparable with any standard lead. By offering diverse possibilities for positioning, it also provides the opportunity for diverse close views of the heart activity. For example, some positions are better for monitoring atrial activities, like the morphology of the P wave. Considering that the ECG body sensors are still novel development, their positioning on the body is still not standardized. Nevertheless, two often used positions are shown in the left and the right part of Figure 6, here named horizontal and vertical position, respectively. If the sensor cannot be positioned on those positions because of anatomical or other reasons, like excessive hairiness or a proximity of a surgery wound, modified positions obtained by translation and rotation of the sensor, as described in [9], can be used. Raw ECG signals recorded from the sensor in nine positions, obtained by translating the sensor from the horizontal position, are shown in Figure 7. The ECG sensor position, in which the measurement is obtained, is graphically presented in the lower right corner of each graph. The recordings demonstrate the remarkable potential of the ECG sensor to reliably detect all the characteristic ECG waves (P, QRS, T) in all positions. Nevertheless, the ECG signals obtained in positions 5, 7, and 8 have the largest amplitudes. All positions, except position 3, also clearly capture the P wave. Figure 8 shows four ECG signals recorded with the ECG sensor in four positions, obtained by rotating the sensor from the vertical position. Again, it can be noted that all the characteristic ECG waves are clearly visible in all four positions. For better detection of the propagation direction of depolarization waves, two or more sensors on different positions can be used for simultaneous ECG measurements.

**Figure 9:**
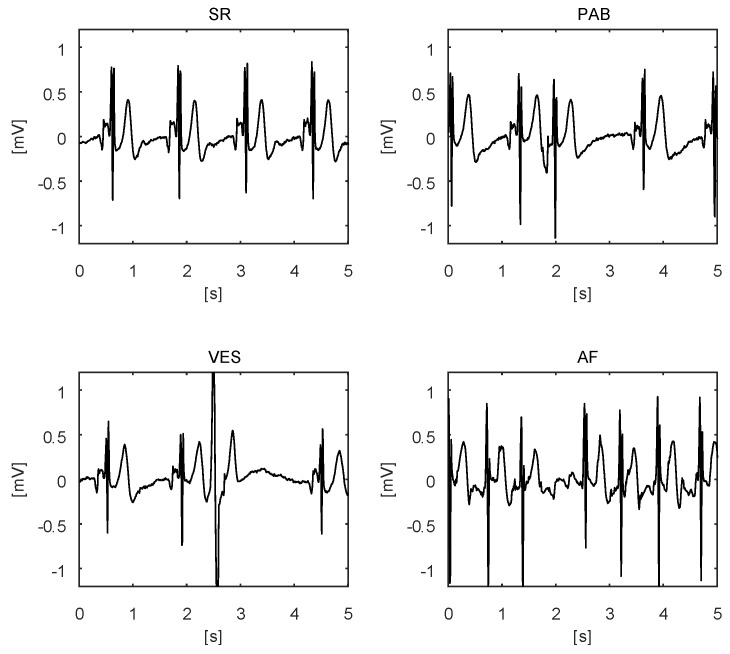
Examples of ECGs with sinus rhythm (**above left**), premature atrial beat (**above right**), ventricular extra systole (**below left**) and atrial fibrillation (**below right**).

To demonstrate the measurement performances of the differential ECG sensor, we have extracted four short segments of raw ECG signal measured on a single subject, with the sensor in position 5 from Figure 7. This position also corresponds closely to the electrodes of the standard leads V1 and V2. The measurements are shown in Figure 9 and they include examples of a sinus rhythm (SR), premature atrial beat (PAB), ventricular extra systole (VES), and atrial fibrillation (AF). Close inspection of the measurements reveals that we can recognize all characteristic ECG waves in the SR signal and that the beat-to-beat intervals in the SR signal are with similar duration. On the other hand, the duration of the beats in the PAB and the VES examples are different. Moreover, in the PAB beat, the P-wave (the depolarization of the atria) is reversed, and, even more, there is no P-wave visible in the VES beats. However, in the VES beats, we can observe a P-wave near the beginning of the T-wave at about 2.8 s. Finally, in the AF example, no consistent P-waves are visible, and the QRS complexes occur in significantly shorter and varying time intervals, which is consistent with the definition of AF. All these examples clearly demonstrate that the quality of the ECG signal is adequate for clinical use.

Considering the presented single-lead ECG, we may expect a significant amount of artefacts in the recorded ECG because of, e.g., poor or even lost contact between the electrode and the skin, changes in electrode-skin conductivity arising from motion interference during activity, muscular noise, etc. Furthermore, the ECG measurements may be easily interrupted by the user, e.g., during shower or if the user is filling uncomfortable. Consequently, the long-term measurements would be usually composed of several ECG records that must be correctly aligned in time. The standardized output formats of the recorded ECG should be used, e.g., ISHNE, HL7, with specific additional features, e.g., a special mark for the missing data bit/packet or a tailored data transmission protocol in the case of a wireless design. The above facts and difficulties are new challenges that are not well covered by the existing programs for ECG analysis. New approaches that are able to exploit the large amount of data are needed to accelerate the analysis. The template based approach for the detection and classification of beats in the existing interpretation software should be supplemented with advanced artificial intelligence (AI) approaches for data analytics, which could help in signal denoising and improved ECG interpretation. We have tested several programs, e.g., AMPS CER-S (http://www.amps-llc.com/) and Quick Reader (http://www.holtersupplies.com/) that can accept the ISHNE format for ECG recorded up to one month or more. However, there is the need for an additional sequenced analysis that will be able to accept multiple files, analyze them, possibly in parallel on more computers, and merge their analyses in a single ECG report. Recently, new innovative approaches appear in the field of ECG analysis, for example, the Cardiomatics software (http://cardiomatics.com/) that employs advanced machine learning techniques to provide the maximum possible value out of ECG recordings.

## 4. Studies and Pilots

This section provides an overview of the studies and pilots where the ECG sensor has been used. These include measurements acquired from volunteers, including pregnant subjects, and measurements obtained during exercise. Next are the measurements obtained in veterinary practice, on dogs, cats, and horses. Then, two pilot studies are presented: screening of patients on primary level at the Health Centre Ljubljana and monitoring of atrial fibrillation after surgery at the University Medical Centre Ljubljana. Finally, we also present some promising applications using the ECG body sensors, such as hearth-rate variability biofeedback assessment and biometric authentication. The section discusses in short the results from successful ECG sensor applications previously published in referenced studies. More details about the studies/pilots can be found in the corresponding references.

### 4.1. Abdominal Fetal ECG

In the years 2015–2017, 67 people volunteered to test the body ECG sensor, either from curiosity to see how it works in everyday life or were suspicious about their heart condition [22]. The total measurements collected were 530 days. The ECG system was attractive for the users since it allowed them to see their ECG measurements online on a smartphone display. The measurements include examples of, among others, atrial fibrillation and arrhythmia during sleep. Among the volunteers, there were also pregnant subjects on which abdominal ECG (AECG) measurements were performed. The AECG can be used as a non-invasive method for monitoring the cardiac activity of a fetus [23].

**Figure 10:**
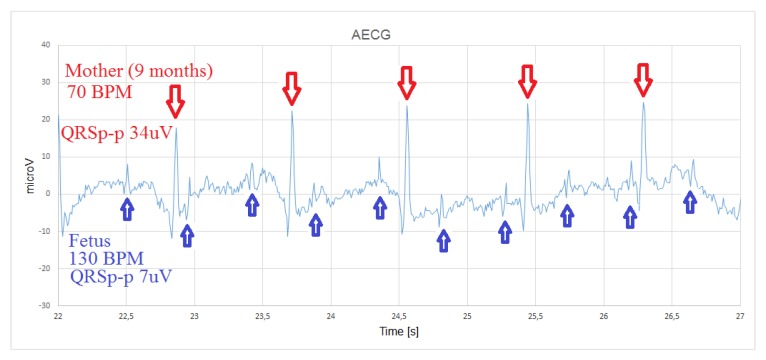
Abdominal ECG recorded in the ninth month of pregnancy with the sensor positioned in the center of the abdomen, 5 cm below the umbilicus.

An AECG measured on a pregnant subject in the ninth month of pregnancy is shown in Figure 10. The recordings demonstrate the remarkable potential of the ECG body sensor for AECG measurements and detection of the fetal heart rate. During recording, the mother was resting in supine position and the sensor was placed in the center of the abdomen, 5 cm below the umbilicus. For effective measurement, it was recommended that the sensor is shield by covering it with both hands to avoid electrical interference. Consequently, the interference from the power grid is not present in the signal, which is crucial for further analysis. The gain of the input signal amplifier was increased for a factor of 12, compared to the production version of the sensor. The raw signal is sampled at a frequency of 125 Hz and a resolution of 0.491 μV. The fetal ECG with heart rate of 130 beats per minute (BPM) is superimposed on the mother’s AECG with heart rate of 70 BPM. Fetal ECG measured on the abdomen during pregnancy has the amplitude significantly lower than the amplitude of the mother’s AECG, which is already of low amplitude, compared to normal differential ECG signals. The AECG peak-to-peak QRS amplitude is approximately 34 μV, while the QRS amplitude of the fetal ECG is about 7 μV. Therefore, the recording can only suffice for the detection of the heart rate.

### 4.2. Sports/Fitness Measurements

**Figure 11:**
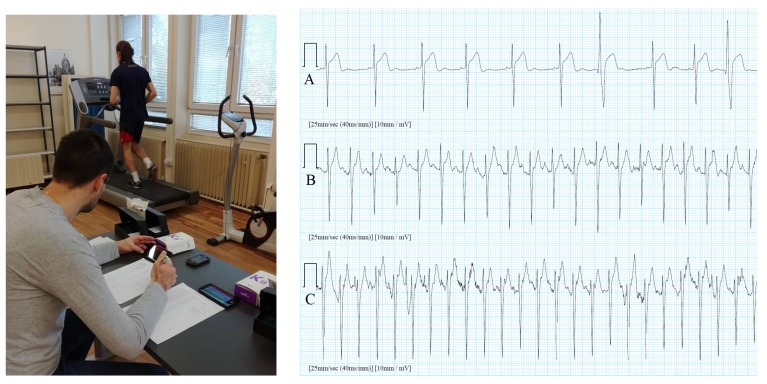
Running on a treadmill with the ECG body sensor fixed with self-adhesive tapes (**left**). Corresponding sections of ECG signals for different running speed (**right**): (**A**) initial resting (heart rate: 74 BPM), (**B**) medium activity (running speed: 14 km/h, heart rate: 140 BPM), and (**C**) maximum activity (running speed: 19 km/h, heart rate: 198 BPM).

Modern professional sport demands extreme efforts from the athletes, and these efforts can present a high risk to their health. Studies have reported the increased risk of cardiovascular events and sudden death during intense exercise—particularly in competitive sports [24]. Significant advances in the prevention of cardiovascular accidents would become possible if the ECG could be measured during regular sports activities. The study in [25] was testing the applicability of ECG measurements for rhythm monitoring during intensive activity, i.e., cycling on ergometer and running on a treadmill according to the RAMP protocol [26]. Two different positions and two different fixation methods of the electrodes have been tested on 23 participants. We have been focused on the evaluation of ECG distortion levels regarding sensor position and its fixation method. Namely, in such cases, the noise of the ECG signal is the highest because of the intensive muscle activity [9] and because of the excessive accelerations when running, which produce sensor movements and consequently artefacts in the measured ECG. Unfortunately, such artefacts exhibit a similar frequency spectrum as the heart rhythm, which additionally complicates the denoising process of the ECG. An example of running on a treadmill with a sensor in position 8 (as shown in Figure 7) and fixed with additional self-adhesive tapes is shown in the left part of Figure 11. Corresponding ECG signals for different activity levels: initial resting, medium activity (running speed 14 km/h), and maximum activity (running speed 19 km/h) are shown in the right part of Figure 11. On the cycle ergometer, the cycling starts with a load of 50 W for females and 75 W for males, and is increased by 25 W after each minute, until exhaustion, i.e., maximal intensity. Based on the obtained results, we have concluded that the ECG signal, measured with the body sensor and standard ECG self-adhesive electrodes, is acceptable (HR is still-assessable) up to a maximal intensity in cycling. In running on a treadmill, the ECG signal was acceptable in average up to 90% of maximal speed, if the sensor is fixed with a self-adhesive tape, and up to 78% of maximal speed in a 100 m sprint outside in the field, if measured with electrodes from the Polar chest belt. The results indicate that ECG body sensors can be used for rhythm monitoring in laboratory and field tests, but attention should be paid to the sensor position and fixation method.

**Figure 12:**
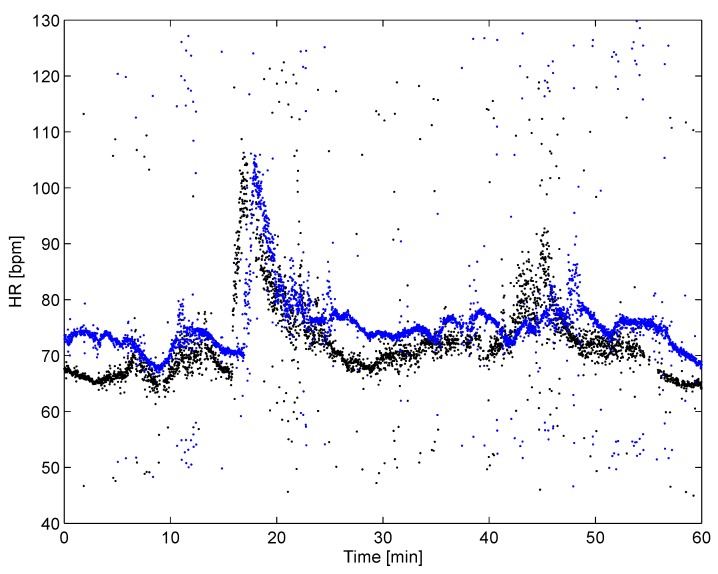
Heart rate (HR) of a study participant during gymnastics in two periods: September 2017 (black) and November 2017 (blue).

Physical inactivity is a global phenomenon, with estimates of one in four adults not being active enough [27], and the consequences of physical inactivity cannot be disregarded, with the inactive population facing a heightened risk of developing chronic degenerative diseases such as cardiovascular disease, cancer, respiratory diseases, and diabetes. The application of unobtrusive body sensors in evidence-based evaluation of the impact of physical activity on the health state of the older population is presented in [28]. The aim of this study was to develop the aforementioned evaluation methodology for elderly people, using questionnaires, measurement of ECG by the presented wearable body sensor, and fitness tests. The volunteering participants have been recruited from the elderly who regularly take part in daily half an hour long open-air exercise entitled “1000 movements”. The ECG body sensor was accepted by the participants and successfully used in the acquisition of the ECG signal during the exercises. Two heart rate (HR) curves of a study participant obtained from ECG body sensor measurements during morning gymnastic, in September 2017 and November 2017, are shown in Figure 12. Dots represent actual HR, i.e., $60/(t_{i}-t_{i-1})$, where $t_{i}$ and $t_{i-1}$ are times of the current beat and the previous beat in seconds, respectively. Note that some dots, far-out from the HR curve, indicate arrhythmic beats. Comparing both HR curves, we observe that the HR before gymnastics was 66 BPM (September) and 74 BPM (November). Then, the HR starts to rise sharply to the peak level of about 106 BPM, in both measurements. After that, the HR is slowing down with slightly different rates to about 70 BPM. Finally, the HR stabilizes at a value close to the initial HR. Further analysis by a trained expert could result in an evidence-based assessment of gymnastics impact on the cardio-vascular system.

### 4.3. Veterinary Medicine

With collaboration between the Jožef Stefan Institute, Ljubljana and the Veterinary Faculty, University of Ljubljana, several studies were conducted demonstrating the usefulness of the ECG sensor in veterinary medicine. First, a comparison of wireless ECG and standard ECG in dogs was made in a pilot study with eight hospitalized dogs with suspected arrhythmias [29]. All arrhythmias documented with the standard ECG were also documented with the wireless ECG sensor. The study proved that wireless ECG monitoring can give satisfactory ECG recordings, regardless of the sensor position, or physical activity, and size of the dog. Next, a case report of a long-term ECG monitoring on a dog with dilated cardiomyopathy was presented in [30]. The ECG sensor was used to record more than 500 h of ECG data over a period of six months. Long-term ECG monitoring was instrumental for excluding arrhythmias as the cause of the dog’s apparent debilitating condition. This enabled a more confident approach to the treatment.

In a broader study, a diagnostic utility of electrocardiogram data has been obtained by monitoring 36 dogs and four cats with suspected arrhythmias with the wireless ECG sensor [31]. For comparison with standard ECG recordings, a combination of 30-min and 24-h ECG recordings was made. When compared to the standard electrocardiogram, equivalent results were obtained when observing either heart rate or duration of the main ECG waves. In 15 animals (37.5% of all animals or 50% of animals with arrhythmia), extension of the ECG monitoring time increased the diagnostic yield; with the wireless device, more arrhythmias were detected than with the standard ECG.

**Figure 13:**
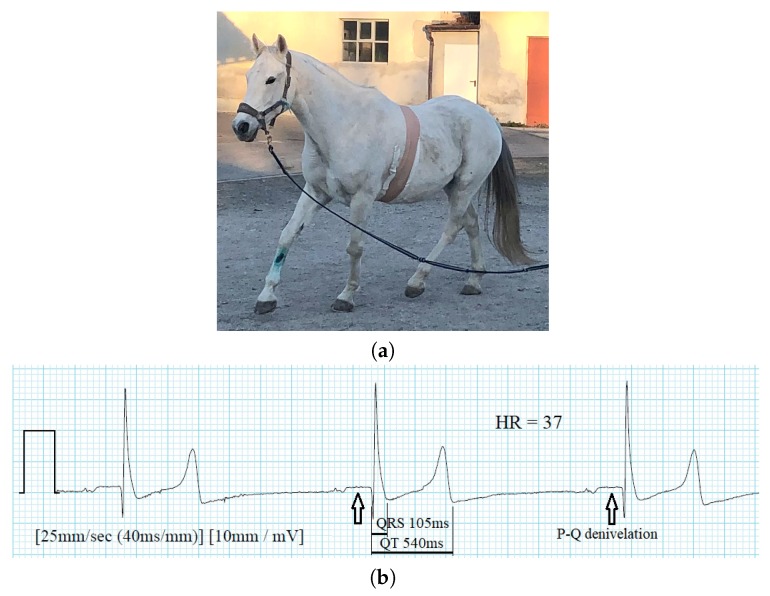
Wireless ECG body sensor used for monitoring in horses. (**a**) Arrab/Lipizanner during exercise. The ECG sensor was bandaged to the thorax to prevent electrode detachment. (**b**) ECG of an adult horse. Note the high amplitude of the QRS compex (2.2 mV) and a typical heart rate while the horse is standing (37 BPM).

Finally, the wireless ECG sensor was used in a study of seven horses performing various activities, i.e., standing, walking, and trotting [32]. The research question was: How does the ECG sensor, which was designed for use on humans, perform on horses? The position of the electrodes was determined experimentally, to obtain the maximal amplitudes of the ECG waves (P, QRS complex, T) and considering the quality of the recording during movements (walking, trotting). The study showed that the wireless ECG sensor can be used for long-term ECG monitoring in horses, monitoring of hospitalized horses, and for monitoring during anesthesia. In Figure 13, the use of the wireless sensor for ECG monitoring in an adult horse is presented. The ECG recording shows high amplitude of the QRS complexes (2.2 mV) and slow heart rate of 37 BPM, which is typical while the horse is standing. Although the horse’s heart is more than 10 times larger than the human’s, the QRS interval is still short (105 ms) and the QT interval is also short (540 ms).

### 4.4. Pilots

Rhythm disorders are often present in patients visiting general physicians. A pilot study for screening patients with a suspicion of irregular heartbeat was started in October 2016 in the Health Centre Ljubljana. Here, we present the study in short, while the details are available in [33]. The goal of the study was to obtain an insight into the practical use of the wireless ECG sensor in patients complaining about heart rhythm disorders. Out of 110 patients enrolled in the study, 100 patients used the ECG sensor. The results showed that 39.3% of the patients had benign rhythm disturbances, 1% had paroxysmal atrial fibrillation, 13.1% had rhythmic ventricular disorders, and 30.3% had anxiety and panic disorder. Based on the results of the recordings from the ECG sensors, the physicians decided to follow-up with 63.5% of the patients, additional diagnostic testing was done in 6.7%, a new medication was prescribed in 6.7%, and 18.3% of the patients were referred to a cardiologist. The conclusion of the study was that a personal ECG sensor with a simplified interpretation of the measurements could introduce new pathways in the healthcare of patients with cardiovascular diseases.

A prospective study of atrial fibrillation was conducted at the Clinical Department of Cardiovascular Surgery, University Medical Centre Ljubljana, from March to July 2018 [34]. Atrial fibrillation is a typical complication after a heart surgery. To date, atrial fibrillation most commonly occurs on days 2 and 3 after the surgery. It occurs more frequently after re-operation due to complications, prolonged ventilation, or re-intubation. One of the goals of the study was to determine if continuous single-channel wireless ECG monitoring from day 1 to day 5 after the operation detects more episodes of the atrial fibrillation than the existing current clinical protocols. Out of 47 patients, 13 developed paroxysmal atrial fibrillation. All 13 cases of AF were detected with the wireless ECG sensor, while clinically established protocols recorded only 9 cases of AF.

### 4.5. Hearth Rate Variability Biofeedback Assessment and Biometric Authentication

Unobtrusive body sensors for long-term measurements enable several new fields of investigation because of their simplified measurement methodology and the ability to introduce big data analytic approaches for the measured ECG streams. Such examples present the studies for hearth rate variability (HRV) biofeedback assessment and biometric authentication using our wearable ECG body sensor.

Using a single ECG sensor, the heart rhythm and its variability can be accurately determined. HRV indices are becoming an important tool in the evidence-based evaluation of different rehabilitation treatments. Recently, an increased number of scientific studies on the impact of biofeedback training on the HRV were published. It is assumed that an increasing HRV indicates an improvement in the rehabilitation of patients following a heart surgery, or other somatic and psychiatric illnesses. Our preliminary research on rehabilitation techniques for increasing the HRV indicates that long-term ECG sensor measurements enable the study of the long-term impact of rehabilitation procedures [35]. Analysis of HRV is also interesting in other practical fields outside of medicine. The impact of noise, temperature, and other environmental influences on subjects can be assessed by HRV. The ECG body sensors are an important tool in such investigations because of their simplicity, low cost, and ability to use them during every day work and activities.

Biometric authentication is one of the promising options where ECG data from wearable body sensor can be exploited. The ECG can serve, besides for its principal purpose of monitoring heart rhythm, as a biometric trait due to its unique identity properties, including user-specific deviations in ECG morphology and heart rate variability. In a preliminary small study [36], we have tested the hypothesis if long-term ECG data, acquired by our unobtrusive chest-worn ECG body sensor, can be applied for accurate user authentication and identification. A novel framework for wearable ECG-based user recognition is proposed, based on higher-order statistics on cyclostationary data. Similar approaches have been already efficiently applied for inertial-sensor-based gait recognition [37]. Experimental ECG data were collected by four subjects during their regular daily activities with more than six hours of ECG data per subject. Preliminary results of the proposed methodology provide error rates from 6% to 13%, depending on the subject. It is evident that further work is needed towards the development of accurate and robust methodology for the recognition performance, e.g., the examination of the influence of HRV and heart anomalies, the influence of sensor position variability, etc. Furthermore, extensive experimental measurements on a much larger set of participants are necessary, both with regular and irregular heartbeats during longer time frames—in terms of months.

## 5. Conclusions

The need for reliable monitoring of the hearth rhythm during every-day activities has motivated the development of devices for long-term ECG monitoring. In this paper, we present the notion of a differential lead and the possibility to implement it in a form of a wireless ECG body sensor. In particular, we describe a conceptual hardware and firmware design of an ECG body sensor, which was developed, produced, and certified for selling on the EU market. We also present our experience regarding the technical aspects of the sensor development, and, even more, the experience obtained from experimental and practical measurements with the ECG sensor. They all confirm that the wearable body ECG sensor is a feasible solution for reliable and accurate long-term heart rhythm monitoring in various aspects of everyday life, not only in humans, but also in animals. Compared to the current standards for ECG monitoring used in medicine, like the 12-lead ECG for monitoring in a hospital environment and the Holter monitor for outside of hospitals, the ECG sensor provides non-obtrusive ECG monitoring during every-day activities for longer periods of time. Although a wearable ECG body sensor carries less information than the standard 12-lead ECG, it can provide various closer looks at the heart activity. Even more, the measurements from several ECG sensors performed simultaneously can provide redundancy and even synthesis of the standard ECG if at least three sensors are placed on appropriate positions on the body. We believe that, in the future, the obtained knowledge with the medical-grade ECG sensor for long-term monitoring will contribute to the development of diagnosis of various heart abnormalities.
